# A practical approach for implementation of a basal-prandial insulin therapy regimen in patients with type 2 diabetes

**DOI:** 10.1186/1750-4732-1-9

**Published:** 2007-04-20

**Authors:** Steven Edelman, George Dailey, Thomas Flood, Louis Kuritzky, Susan Renda

**Affiliations:** 1 University of California San Diego, Veterans Affairs Medical Center, San Diego, CA, USA; 2 Scripps Clinic, La Jolla, CA, USA; 3 Georgia Center for Diabetes, Atlanta, GA, USA; 4 University of Florida College of Medicine, Gainesville, FL, USA; 5 Clinical Associates, Reisterstown, MD, USA

## Abstract

Basal-prandial insulin therapy is a physiologic approach to insulin delivery that utilizes multiple daily injections to cover both basal (ie, overnight fasting and between-meal) and prandial (ie, glucose excursions above basal at mealtime) insulin needs. While basal-prandial therapy with multiple daily injections is an important therapeutic option for patients with type 2 diabetes, there is a common perception that this therapy is difficult to initiate in the primary care setting. To address this issue, a panel of clinical experts convened to develop practical recommendations on how to initiate basal-prandial therapy in patients with type 2 diabetes, focusing on patient selection, simple dosing and titration, and monitoring. Patients with type 2 diabetes who are appropriate candidates for basal-prandial insulin therapy include those who: 1) are unable to achieve glycemic control on oral antidiabetic drugs, 2) are unable to achieve glycemic control on split-mixed/premixed insulin regimens, 3) are newly diagnosed but unlikely to respond to oral antidiabetic drugs alone (ie, the patient has severe hyperglycemia or a markedly elevated glycosylated hemoglobin A1C level for which oral antidiabetic drug therapy alone is unlikely to achieve goals), and 4) prefer this therapy due to socioeconomic or other individual considerations. Basal-prandial insulin can be initiated in a simple stepwise manner, starting first with the addition of basal insulin to the existing oral antidiabetic drug regimen, followed by the introduction of 1 prandial insulin injection to the basal insulin plus oral antidiabetic drug regimen (after basal insulin has been optimized). Subsequently, other injections of prandial insulin may be added when needed. Based on home glucose monitoring data, patients may be converted from split-mixed or premixed insulin regimens to basal-prandial regimens with similar ease. Basal-prandial therapy using newer insulin formulations, such as long- and rapid-acting insulin analogs, can be relatively simple to use in patients with type 2 diabetes and is an appropriate methodology for application by primary care clinicians.

## Background

Diabetes has reached epidemic proportions in the United States, with an estimated 20.8 million people (>6% of the population) affected by the disease and 13 million patients have been diagnosed [1,2]. An estimated 90%–95% of cases are type 2 diabetes [2]. Despite abundant evidence regarding the increased risk of serious micro- and macrovascular complications associated with poor glycemic control, only a small proportion of patients with type 2 diabetes attain the recommended treatment goals (Table 1). The 2007 American Diabetes Association (ADA) Clinical Practice Recommendations specify a glycosylated hemoglobin A1C (hereafter A1C) target of < 7.0% for glycemic control [3]. This recommendation is based on extensive epidemiologic data, [4,5] and recent clinical studies demonstrate that patients with type 2 diabetes can achieve glycemic targets [6]. However, the National Health and Nutrition Examination Survey 1999–2000 showed that only 37% of adults with diabetes are achieving the target A1C level of < 7.0% [7]. One important reason for this is failure to appropriately initiate insulin therapy in a timely manner [8].

**Table 1:** ADA 2007 Treatment Recommendations [3]

**Glycemic control:**	
A1C	< 7.0%*^†^
Preprandial glucose or FPG	90–130 mg/dL
Peak postprandial capillary plasma glucose	< 180 mg/dL
**Keypoints:**	
• Goals should be individualized	
• Special populations may require treatment modifications	
• If A1C goals are not met despite reaching preprandial glucose goals, target treatment to PPG goals if home glucose monitoring data demonstrate abnormally high blood glucose levels	

*Referenced to a nondiabetic range of 4.0%–6.0% using a Diabetes Control and Complications

Trial-based assay.

^†^More stringent glycemic goals (ie, a normal A1C < 6.0%) may further reduce complications at the cost of increased hypoglycemia risk.

Copyright ^© ^2007 American Diabetes Association from Diabetes Care, Vol. 30, 2007; S4-S41 Modified with permission from *The American Diabetes Association*

The United Kingdom Prospective Diabetes Study demonstrated the progressive decline in β-cell function that occurs over time in type 2 diabetes and the eventual need for insulin therapy in most patients [9,10]. Early introduction of insulin therapy can attain and maintain glucose targets when oral antidiabetic drug (OAD) regimens have failed to achieve glycemic goals, thereby reducing the risk of diabetes-related complications [11]. Recent consensus conference recommendations from the American College of Endocrinology indicate that glycemic targets can be effectively achieved by basal insulin plus an OAD or basal-prandial insulin regimens in type 2 diabetes [12].

Basal-prandial insulin therapy with multiple daily injections is a physiologic approach that attempts to approximate the normal pattern of pancreatic insulin secretion [13,14]. Basal insulin suppresses glucose production by the liver (gluconeogenesis) between meals and overnight [13,15,16]. Prandial (bolus) insulin covers increases in blood glucose levels following meals [13]. The combination of basal and prandial therapy is an important option for patients with type 2 diabetes when glycemic control is not achieved with OADs alone or basal insulin plus OAD therapy [14]. Although primary care clinicians may consider referral of patients considered for basal-prandial insulin regimens, this strategy can be appropriately implemented within most primary care offices.

Basal-prandial therapy is underutilized in the primary care setting because it is often perceived as being complex, difficult to implement, time-consuming, and/or reserved mainly for patients with type 1 diabetes. However, insulin therapy can be implemented in a simple, stepwise manner, by first introducing basal insulin and subsequently adding prandial insulin (starting with the largest daily meal and advancing over time to the other 2 meals) as needed based on pre- and postprandial blood glucose monitoring. This review provides practical recommendations for initiating basal and basal-prandial insulin therapy in type 2 diabetes, with a focus on insulin analogs. The recommendations were developed during a recent meeting of clinical experts in the field of diabetes. The meeting was supported by an educational grant from sanofi-aventis and focused on practical aspects of initiating insulin therapy including patient selection, dosing, dose titration, and monitoring (a reference guide on how to initiate basal-prandial insulin has been developed by this group and is available as an online resource) [17].

## Rationale for Basal-Prandial Insulin Therapy

Both basal and postprandial glucose (PPG) excursions contribute to hyperglycemia in type 2 diabetes, and treatment strategies that address both components may enhance attainment of glycemic goals. The ADA guidelines call for a fasting plasma glucose (FPG) target of 90–130 mg/dL and a 2-hour PPG target of < 180 mg/dL (Table 1).

Failure to achieve glycemic control can lead to the development of serious diabetes-related complications [4,5]. There is also a growing body of evidence indicating that postprandial hyperglycemia independently contributes to an increased risk for macrovascular complications [4,5,15,16,18-21]. In fact, a meta-analysis of the data has indicated that isolated postprandial hyperglycemia (2-hour PPG >140 mg/dL) in the presence of normal FPG (< 110 mg/dL) and normal A1C (< 6.1%) is associated with a 2-fold increase in the risk of death from cardiovascular disease [15,21].

**Figure 1:**
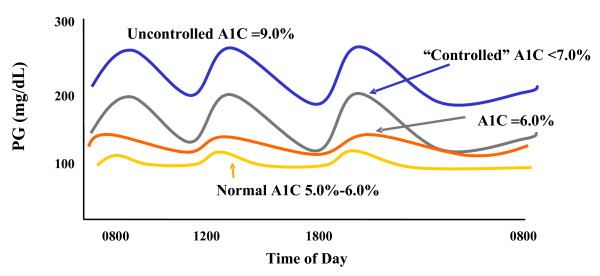
**24-hour glucose profiles for representative patients at different levels of glycemic control**. Increasing A1C values reflect an elevated fasting or preprandial (basal) blood glucose level and elevated PPG excursions. At levels shown as "uncontrolled" A1C (9.0%), the culprit is predominantly loss of control of the FPG, whereas the difference between an A1C of upper normal (6.0%) vs "controlled" A1C (7.0%) predominantly reflects increased PPG. (PG = plasma glucose.) Copyright ^© ^2002 From Rationale for and strategies to achieve glycemic control by Cefalu WT. In: Leahy JL, Cefalu WT (eds) *Insulin Therapy*. Reproduced by permission of Routledge/Taylor & Francis Group, LLC [41].

A stepwise approach to basal-prandial insulin therapy in patients with type 2 diabetes allows treatment to be advanced as the disease progresses to minimize the risk of complications. Initially, elevated baseline FPG levels lead to a higher overall plasma glucose profile and consequently higher PPG excursions [16,22]. Also, the relative contribution of FPG to A1C progressively increases as glycemic control worsens [16,22]. The first goal of a physiologic insulin therapy regimen, therefore, is to lower the overall glycemic profile with basal insulin and normalize, or nearly normalize, the FPG level [14]. Conceptually, "fix the fasting first" is the initial agenda. This is accomplished most efficiently by the addition of basal insulin. Decreases in PPG excursions may also be achieved by lowering the overall glycemic profile with basal insulin (Figure 1). However, as the disease progresses, the capacity of patients with type 2 diabetes to respond to PPG excursions becomes progressively impaired due to reduced endogenous insulin secretion resulting from loss of β-cell function [9,16]. In many patients, basal insulin alone, when added to OADs, is sufficient to attain glucose goals. Due to the natural history of type 2 diabetes, many patients eventually progress to a level of insulin deficiency that requires initiation of prandial insulin (or pharmacotherapy targeted to prandial insulin control) in addition to basal insulin. Prandial insulin can be added with the appropriate meal or meals to control PPG excursions.

## Treatment Guidelines

**Figure 2:**
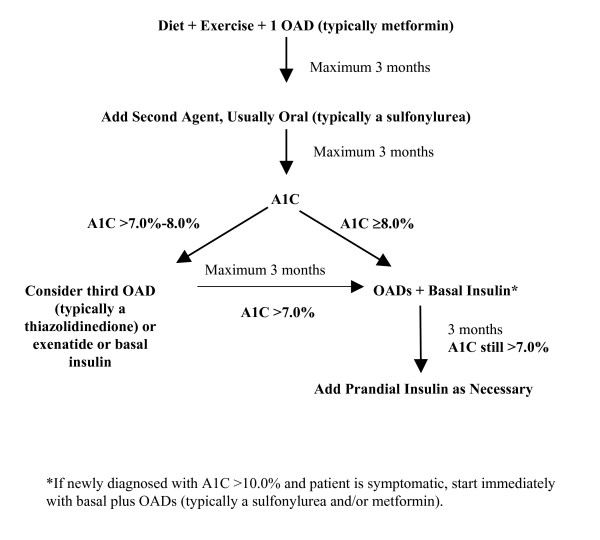
Type 2 diabetes treatment algorithm.

For patients with type 2 diabetes, A1C values ideally should be measured every 3–6 months. Adjust therapy when glycemic control is above the ADA target of 7.0% (Figure 2) [3]. In type 2 diabetes, a progressive decline in β-cell function should be anticipated and appropriate adjustments to therapy made whenever glycemic goals are not maintained [23].

## Practical Recommendations for Patient Selection

A combination of basal insulin with OADs or basal-prandial insulin therapy are both appropriate treatment options for patients with type 2 diabetes who are:

• Newly diagnosed and unlikely to achieve glycemic control with OADs alone (eg, a patient who presents with markedly elevated A1C [ie, 10.0%]) [24]

• Unable to achieve and/or maintain optimal glycemic control on diet and exercise plus OADs

• Unable to achieve and maintain glycemic control on split-mixed or premixed insulin regimens.

Insulin-naive patients who are unable to achieve or maintain glycemic goals on OADs can advance therapy to basal insulin plus OADs then basal-prandial therapy in a stepwise manner, starting first with the addition of basal insulin to the existing OAD regimen. Prandial insulin is added to the regimens of patients not achieving glycemic goals despite well-controlled fasting blood glucose after 3–6 months of basal insulin therapy (Figure 2) [14]. Initially, prandial insulin therapy may only need to be provided with the largest meal of the day, or whichever meal produces the greatest glucose excursions from baseline.

Certain patients with newly diagnosed type 2 diabetes may benefit from early initiation of basal-prandial insulin therapy, including those with glucose toxicity (ie, prolonged hyperglycemia leading to impaired glucose disposal and impaired glucose-stimulated insulin secretion by β-cells) or latent autoimmune diabetes of adulthood (LADA). LADA is caused by immune-mediated destruction of the insulin-producing pancreatic β-cells, similar to type 1 diabetes but typically is diagnosed in patients aged 30–40 years (the diagnosis is confirmed by blood tests for the presence of glutamic acid decarboxylase antibodies) [25,26]. Newly diagnosed patients with A1C >10.0% require more than a 3.0% reduction in A1C to achieve ADA target glucose levels [3]. Reductions in A1C of this magnitude generally will not be achieved with OADs alone, especially in the face of glucose toxicity, thus such patients who are symptomatic should be started on basal-prandial insulin immediately. Once insulin has successfully reversed the glucose toxicity, many of these newly diagnosed patients can then be controlled on OADs [2,12]. Notably, patients with LADA generally do not respond significantly to OADs and will require insulin therapy at an earlier stage than other patients with type 2 diabetes [25-27].

Finally, patients with type 2 diabetes on split-mixed or premixed twice-daily insulin regimens who are unable to achieve or maintain target A1C goals may also benefit from conversion to basal-prandial insulin regimens [14]. Because the timing of the dose is fixed in such regimens, they may be ineffective in patients with an unpredictable daily routine [14]. Patients on these regimens can be directly converted to a basal-prandial approach using the protocol described below. In addition, because patient reluctance to use needles has already been overcome in this group, it might be easier to transition patients to multiple daily injections, if necessary.

## Preparing the Patient for Basal-Prandial Insulin Therapy

Clinicians can prepare patients for basal-prandial insulin therapy by facilitating discussions about insulin and educating patients on aspects of diabetes self-management, including the importance of diet and exercise, injection techniques, carbohydrate counting, home glucose monitoring, and hypoglycemia awareness (Table 2). Above all, it is important that the clinician project a positive attitude about insulin when discussing this therapy with the patient [8]. Clinicians should avoid using insulin as a means for threatening or blaming patients for previous treatment failure, but rather explain that insulin therapy is often a natural consequence due to the progressive nature of the disease: nearly one third of patients with type 2 diabetes are likely to require insulin at some point [8]. It is also important that clinicians discuss and come to an agreement with patients on specific treatment goals for basal-prandial insulin therapy [13]. If OADs have failed due to gross dietary noncompliance, addition of insulin therapy is unlikely to achieve glycemic goals without adequate diabetes education. Explaining the significance of A1C measurements, discussing A1C goals, and sharing results is an important motivational tool for patients on insulin therapy [8].

**Table 2:** Preparing the Patient for Insulin Therapy

• Discuss insulin at time of diagnosis of diabetes
• Dispel myths about insulin

• Maintain a positive attitude [8]
- Assure the patient that the need for insulin does not represent a personal failure on his or her part
- Express confidence in the patient's ability to master self-injection techniques and to maintain appropriate schedules

• Discuss treatment goals with patient [8,43]
- Explain the rationale for adding insulin to the treatment regimen and the health benefits associated with improved glycemic control
- Allay patient fears about possible negative health consequences associated with insulin therapy
- Assure the patient that the need for insulin does not mean his or her diabetes has worsened to a point where it cannot be managed successfully

• Discuss the patient's day-to-day routine and habits [8]
- Identify how food, exercise, and lifestyle choices may influence therapy and treatment goals
- Assure the patient that he or she can continue to take part in favorite activities, including eating in restaurants and travel

Home glucose monitoring is crucial and should be employed at some level by all patients with type 2 diabetes to provide feedback on both glycemic control and hypoglycemia [3]. For patients taking multiple insulin injections the frequency of home glocose montiring should be at least 3 times daily. While there isn't a specific guideline for patients on less frequent or no insulin, glucose monitoring is useful in helping achieve glycemic goals and should be used with increased frequency whenever modifications are made to the diabetes regimen. Patient education on the target range for glucose values, glucose levels indicating actual or impending hypoglycemia, and appropriate insulin dose adjustment should be reinforced. Ideally, patients should have access to a certified diabetes educator, instructional classes, and/or support groups that can instruct them on home glucose monitoring. It is essential that patients understand the importance of reporting home glucose monitoring results to the clinician and are encouraged to do so (ie, via email, fax, phone, in person). This will enhance the clinician's ability to assess the patient's progress and make timely dose adjustments. Importantly, patients will be more informed participants in their own care.

Although severe hypoglycemia is relatively rare in type 2 diabetes, even among patients on insulin therapy, these events can occur [8]. It is important to educate patients about symptoms associated with hypoglycemia (Table 3) and common causes, including failure to eat after taking insulin, overaggressive use of insulin (ie, increasing the insulin dose in response to an anomalous blood glucose concentration), overexercising without adjusting the insulin dose, and alcohol consumption [8,28]. Patients and family members also need education on how to treat hypoglycemia (eg, with simple carbohydrates).

**Table 3:** Symptoms Associated With Hypoglycemia

**Hypoglycemia**	**Nocturnal Hypoglycemia**
Hunger	Nightmares
Excessive perspiration	Night sweats
Confusion	Tired upon awakening
Difficulty speaking	Irritable upon awakening
Nervousness	Confused upon awakening
Anxiety	
Dizziness	
Shakiness	
Weakness	
Sleepiness	
Increased heart rate	
Visual disturbances	

## Available Insulin and Other Injectable Preparations

Various insulins with different time-action profiles are currently available and can be used as part of a basal-prandial insulin regimen (Table 4). Currently, the intermediate-acting neutral protamine Hagedorn (NPH) insulin and the long-acting insulin analogs, insulin glargine and insulin detemir, are available for basal insulin replacement [13,14,29]. Several insulin preparations are now available for prandial coverage, including the short-acting regular human insulin, the recently available inhaled insulin, and the rapid-acting analogs, insulin lispro, insulin aspart, and insulin glulisine [13,14,30].

**Table 4:** Time-Action Profiles of Insulins [44]

Insulin Type	Onset	Peak (h)	Duration of Action (h)
Rapid acting			
Lispro, aspart, glulisine	5–15 min	0.5–1.5	2–4
Inhaled insulin [32]	5–15 min	0.5–1.5	3–6
Short acting			
Regular human	30–60 min	2–3	3–6
Intermediate acting			
Human NPH	2–4 h	4–10	10–16
Long acting (basal)			
Insulin glargine	1–2 h	No pronounced peak	≈ 24
Insulin detemir [45]	1–2 h	Less pronounced peak (≈ 6)	18–24

Premixed:

Comprised of intermediate-acting insulin with either regular human insulin or a rapid-acting analog in a fixed ratio (eg, 70/30); thus, the onset, peak, and duration reflect the combined effect of these components.

Copyright ^© ^2002 From Insulin therapy in type 2 diabetes mellitus by Ahmann AJ, Riddle MC. In: Leahy JL, Cefalu WT (eds) *Insulin Therapy*. Reproduced by permission of Routledge/Taylor & Francis Group, LLC

**Figure 3:**
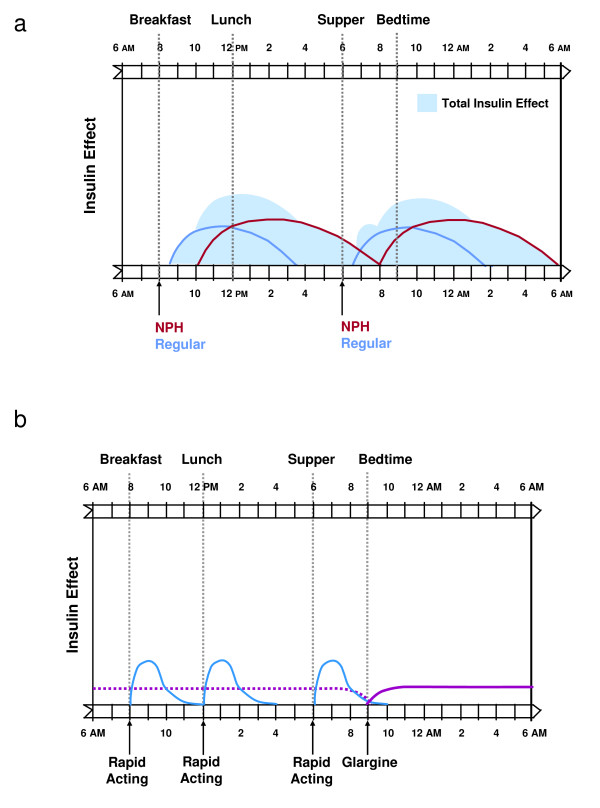
**Basal-prandial insulin replacement profiles using (A) NPH plus regular human insulin and (B) insulin glargine plus a rapid-acting insulin**. Reprinted with permission from DeWitt DE et al. *JAMA *2003, **289:**2254–2264 [14].

While use of human NPH insulin has been the mainstay approach for basal insulin therapy for many years, NPH often does not adequately and consistently provide 24-hour basal insulin delivery, necessitating at least twice-daily dosing in many individuals (Figure 3A) [14,31]. NPH typically has a pronounced peak effect within 4–10 hours of its administration, before returning to baseline within 10–16 hours [13,14,31]. In contrast, insulin glargine is a long-acting basal analog that provides insulin delivery over a 24-hour period without a pronounced peak (Figure 3B) [13,14,31]. In patients with type 2 diabetes, insulin glargine given once daily has been shown to be as effective as NPH for improving glycemic control, while causing significantly less nocturnal hypoglycemia [6]. In a recent study, the basal analog insulin detemir given twice daily showed comparable efficacy to NPH insulin with significantly less nocturnal hypoglycemia [29].

Regular human insulin has traditionally been used to provide prandial insulin coverage [14]. In comparison to insulin analogs, this insulin formulation has a delayed onset of action, requiring it to be administered approximately 30 minutes *before *mealtime in order to match its peak insulin effect to postprandial rises in blood glucose levels [13,14]. Compared to rapid-acting analogs, regular human insulin has less dosing flexibility and a longer duration of action relative to PPG excursions, peaking within 2–3 hours and returning to basal concentrations within 3–6 hours (Figure 3A) [13,31]. This prolonged duration can increase the risk of hypoglycemia if the timing of planned meals is delayed and the patient is on NPH insulin [13,14]. The newer rapid-acting insulin analogs, insulin lispro, aspart, and glulisine, are absorbed more rapidly and are active for a shorter duration than regular human insulin and thus provide a more physiologic profile (Figure 3B) [30,31]. Rapid-acting insulin analogs have an onset of action within 15 minutes of administration, a peak effect within 0.5–1.5 hours, and a return to basal concentrations within 2–4 hours [13]. Because of their rapid onset of action, these insulin analogs should be injected immediately before mealtime [31]. Use of rapid-acting insulin analogs has been associated with improved PPG control and a reduced incidence of severe and nocturnal hypoglycemia, compared with regular human insulin [14]. Inhaled insulin, has a more rapid onset and peak of action, similar to rapid-acting analogs, with a slightly longer duration of action [32].

Other agents for control of PPG include incretin mimetics (eg, glucagon-like peptide-1 [GLP-1] analogs) and amylin analogs. Use of the GLP-1 analog exenatide reduces PPG and results in weight loss, however, it is not currently approved for use with insulin [33,34]. The amylin analog pramlintide is currently approved as adjunctive therapy in patients using mealtime insulin therapy, and use of this drug can result in weight loss as well as improved glycemic control [35].

## Dosing and Titration of Basal-Prandial Insulin Therapy in Insulin-Naive Patients

This section provides practical recommendations for initiating basal and prandial insulin in patients with type 2 diabetes who are not achieving glycemic targets on OADs alone. Common insulin misuse scenarios are detailed in Table 5.

**Table 5:** Practical Recommendations for Optimizing Insulin Use by Primary Care Clinicians

• Avoid delays in the initiation of insulin therapy	• Continue OADs when initiating a basal insulin
• Look for patterns of hyperglycemia when monitoring	• Titrate basal and prandial doses appropriately

### Practical Recommendations for Initiation of Basal Insulin

Insulin-naive patients with type 2 diabetes who fail to achieve or maintain adequate glycemic control on OADs over 3–6 months should be started on basal insulin therapy. In these patients, a single daily dose of basal insulin may be added to existing OADs (continued at the same dosages). Insulin glargine may be safely administered either at bedtime or in the morning. As long as administration is at 24-hour intervals, the time of administration can be tailored for patient convenience or preference. NPH and detemir can be given at bedtime (detemir can be given with the evening meal) but may need to be administered at least twice daily for basal coverage [36]. Based on the panel's clinical experience, basal insulin may be initiated at doses of 10 U in thin patients and 15 U in obese (body mass index >30 mg/m^2^) patients. Following initiation of insulin, patients should monitor FPG levels using home glucose monitoring. Insulin doses can be titrated according to self-monitored FPG values using a titration schedule such as that shown in Table 6[6]. Use of this algorithm in a clinical trial resulted in an insulin glargine dose of approximately 0.5 U/kg of body weight (ie, 50 U in a 100-kg adult). The insulin dose can be actively titrated until the FPG level is ≤ 100 mg/dL, unless there are hypoglycemic episodes. Alternatively, insulin doses may be titrated by the provider or the patient by increasing the insulin dose 2 U every 3 days until FPG levels are ≤ 100 mg/dL [37]. Recent data suggest that less aggressive FPG goals may also be effective as long as close monitoring of glucose and active dose titration of insulin are implemented [38].

**Table 6:** Titration Schedule for Basal and Prandial Insulin [6]

Blood Glucose Levels for 3 Consecutive Days (Fasting, Preprandial, or Bedtime)	Adjust Basal Insulin Dose (U)*	Adjust Rapid-Acting Insulin Dose (U per Injection)
**≥**180 mg/dL	+8	+3
160–180 mg/dL	+6	+2
140–160 mg/dL	+4	+2
120–140 mg/dL	+2	+1
100–120 mg/dL	+1	Maintain dose
80–100 mg/dL	Maintain dose	- 1
60–80 mg/dL^†^	- 2	- 2
< 60 mg/dL^†^	- 4	- 4
Comments:	• For elevated fasting blood glucose levels, adjust only the basal insulin dose	• For elevated preprandial blood glucose at lunchtime, adjust breakfast rapid-acting insulin dose • For elevated preprandial blood glucose at dinnertime, adjust lunchtime rapid-acting insulin dose • For elevated bedtime blood glucose, adjust dinnertime rapid-acting insulin dose

*Copyright ^© ^2003 American Diabetes Association from Diabetes Care, Vol. 26, 2003; 3080–3086. Modified with permission from *The American Diabetes Association*.

^†^If any single blood glucose measurement is in this range, make the appropriate reduction in insulin dose.

Although NPH may have efficacy in achieving glycemic control that is comparable to long-acting insulin analogs, direct comparison trials have shown that rates of overall and nocturnal hypoglycemia are lower with insulin analogs [6,29]. Bedtime dosing of NPH may predispose patients to nocturnal hypoglycemia, while morning dosing of NPH may predispose patients to late-morning and early afternoon hypoglycemia [13].

### Practical Recommendations for Initiation of Prandial Insulin

If A1C goals are not achieved after a period of 3–6 months of treatment with basal insulin plus OADs, patients should be instructed to monitor glucose preprandially and/or 1–2 hours after each meal on a rotating basis to identify the main meal that is contributing to hyperglycemia (ie, high blood glucose levels at breakfast, lunch, dinner, or bedtime). Once identified, 5–10 U of rapid-acting insulin should be administered before this meal. Inhaled insulin is currently available as 1-mg and 3-mg blisters (equivalent to 3 U and 8 U of SC regular human insulin, respectively). If A1C goals are still not reached after 3–6 months of therapy with OADs and basal insulin plus 1 prandial insulin dose at the main meal, prandial insulin can be added to other meals based on home glucose monitoring. For example, if blood glucose levels are high before lunch, add 5–10 U of rapid-acting insulin at breakfast, with continued titration according to the schedule shown in Table 6.

### Implications of Basal-Prandial Regimens for Existing OADs

The OAD regimen should be continued until the addition of insulin achieves glycemic control goals. As glycemic control is established (A1C < 7.0%), OADs can be adjusted in patients on basal-prandial insulin therapy (eg, the sulfonylurea dose can be reduced by ≥ 50% as necessary). If subsequent monitoring clearly shows prompt loss of control, the original dosages of OADs should be resumed. Once prandial insulin is added to basal insulin therapy, secretagogues (eg, sulfonylureas) may be discontinued. Metformin therapy should generally be continued, whereas the decision to continue and/or adjust the thiazolidinedione dose may be left to physician discretion. Typically, if significant glycemic benefit with the OAD was achieved prior to starting insulin therapy, the drug may be continued. In some patients, it may be appropriate also to reduce basal insulin doses after starting prandial insulin with continued monitoring of FPG levels.

## Practical Recommendations for Converting Patients from Split-Mixed or Premixed Insulin to Basal-Prandial Insulin

If split-mixed or premixed insulin regimens fail to achieve or maintain adequate glycemic control, these may be converted to basal-prandial insulin therapy as follows: the total daily dose of NPH insulin (intermediate-acting insulin) should be reduced by 20% in order to calculate the initial dose of the long-acting insulin analog as shown below:

Calculating the initial basal insulin analog dose

1. Determine the total daily dose of NPH

2. Decrease dose of NPH insulin by 20%

Example: A patient on 50 U premixed 70/30 (NPH 35 U; regular human insulin 15 U) twice daily

1. Total daily dose of NPH = 70 U

2. Long-acting analog dose = 80% of 70 U NPH = 56 U

Insulin glargine can be administered once daily either at bedtime or in the morning. Insulin detemir can be administered once daily with the evening meal or at bedtime or twice daily with a morning dose and an evening dose 12 hours later. Following initiation of a basal insulin analog, patients should be instructed to monitor FPG and adjust their dose according to the suggested titration algorithms (Table 6).

A rapid-acting insulin may need to be added to 1 or more meals (eg, at lunchtime since the morning NPH insulin has been eliminated—this can be determined by monitoring postlunch or predinner glucose levels). The dose of rapid-acting insulin can be directly calculated from the dose of regular human insulin:

Calculating the initial rapid-acting insulin dose:

1. Determine the total daily dose of regular human insulin

2. Rapid-acting dose per meal = total daily dose of regular human insulin ÷ 3

Example: A patient on 50 U premixed 70/30 (NPH 35 U; regular human insulin 15 U twice daily)

1. Total daily dose of regular insulin = 15 U twice daily = 30 U

2. Rapid-acting dose per meal = 30 U ÷ 3 = 10 U

NOTE: Pre- and/or postmeal blood glucose should be monitored to assess the need for further dose adjustment.

As is the case with basal insulin, the dose of rapid-acting insulin should be titrated according to the titration schedule shown in Table 6.

### Converting patients to a basal-prandial insulin regimen using a morning dose of a basal insulin analog

The recommended strategy for converting patients to a basal-prandial regimen using morning doses of a basal insulin analog differs slightly from those for patients using bedtime doses. Patients should be given, on the prior evening, a predinner dose of split-mixed insulin (NPH plus regular human insulin) or a predinner dose of premixed insulin as usual. Then they can be administered an initial dose of the basal insulin analog the following morning, with a rapid-acting insulin provided at mealtime(s) as needed.

### Converting patients to a basal-prandial insulin regimen using a bedtime dose of a basal insulin analog

Patients starting basal-prandial insulin therapy with a long-acting analog administered at bedtime should take their morning dose of split-mixed insulin (NPH plus regular human insulin) as usual (they may also take the usual dose of regular human insulin at dinnertime but not a predinner dose of NPH). Patients should then administer the basal analog at bedtime. Starting the following morning, a rapid-acting insulin may be provided at mealtime(s) as needed.

## Practical recomendations for home glucose monitoring

Home glucose monitoring ideally should be performed 3–4 times per day at least initially [3,13]. However, because insurance companies often do not pay for more than 2–3 test strips per day, the following recommendations have been developed as a best practice minimum.

Premeal glucose levels should be tested twice daily before meals and at bedtime. For example, on odd-numbered days of the month, premeal glucose levels should be tested before breakfast and lunch. On even-numbered days of the month, premeal glucose levels should be tested before dinner and at bedtime. Recommended blood glucose targets are 90–130 mg/dL before meals [3] and 100–140 mg/dL at bedtime.

Patients with premeal glucose levels outside the 90–130 mg/dL range may have dietary factors that are impacting their glucose levels or simply require more insulin. Patients should be instructed to take a dietary history to collect additional information on their eating habits and how these habits may be affecting blood glucose levels (ie, snacking between meals). Blood glucose levels may need to be monitored more frequently in these patients over a short period of time, ideally 4 times per day for at least 2 weeks.

Patients with premeal glucose levels within the recommended target range, but who remain above A1C goals, will require even more specific guidance on how to refine glucose control. Hence, to define whether PPG immediately postmeal is the culprit responsible for inadequate control, they should be instructed to specifically monitor 2-hour PPG levels [39]. Another useful monitoring strategy is to have patients "frame" a meal by checking premeal and 2-hour PPG. Patients can choose a different meal each day. Monitoring both premeal and 2-hour PPG may help identify problem meals undetected by premeal glucose monitoring alone. A dietary history should also be taken for these patients. Continuous glucose monitoring is now available and is an important advancement in the management of diabetes since patients will have improved their ability to adjust insulin and to better anticipate and therefore prevent hypoglycemia [40].

## Conclusion

Basal-prandial insulin therapy provides a physiologic approach to the treatment of patients with type 2 diabetes. It can be an appropriate treatment option for a range of patients, including those who are unable to achieve glycemic control on OADs, certain patients with newly diagnosed diabetes and glucose toxicity who are unlikely to adequately attain goals by means of OADs, and patients who are unable to achieve glycemic control on split-mixed or premixed insulin regimens. The availability of newer rapid-acting and long-acting insulin analogs that closely mimic normal endogenous prandial and basal insulin secretion provides an unprecedented opportunity for more physiologic insulin replacement. The proposed approach for dosing, titration, and monitoring of basal-prandial insulin therapy can assist primary care clinicians in initiating and converting patients to basal-prandial insulin regimens consisting of basal insulin and rapid-acting insulin in a straightforward and stepwise manner.

## Competing interests

Steven Edelman, MD, George Dailey, MD, Thomas Flood, MD, and Susan Renda, CRNP, CDE, declare they have no competing interests.

Louis Kuritzky, MD, is or has been on the speakers bureau for Eli Lilly and Company, sanofi-aventis US, and Pfizer Inc.

The expert panel meeting which provided the basis for the recommendations in this article was supported by an educational grant from sanofi-aventis.

## Authors' contributions

Each author was involved in the discussion of the concept of this article, revising it critically for important intellectual content and provided final approval of the manuscript.
